# Influence of Thermally Polluted Water on the Growth of Helophytes in the Vicinity of a Colliery Waste Tip

**DOI:** 10.1007/s11270-012-1323-1

**Published:** 2012-09-22

**Authors:** Damian Chmura, Tadeusz Molenda

**Affiliations:** 1 Institute of Engineering and Environmental Protection, Faculty of Materials and Environmental Sciences, University of Bielsko-Biała, 2 Willowa Str, 43-309 Bielsko-Biala, Poland; 2 Chair of Physical Geography, University of Silesia, Będzińska 60, 41-200 Sosnowiec, Poland

**Keywords:** Leachate, Macrophytes, Wetland, Thermal pollution, Mining waste

## Abstract

The impact of thermal pollution of leachate from a post-coal mine heap on three macrophyte species: *Phragmites australis*, *Typha latifolia*, and *Scirpus sylvaticus* was examined over the entire vegetation season. Hydrological measurements showed that the temperature of the leachate was ca 50 °C at the site of leachate inflow and decreased to ca 15 °C at the end of discharge canal. The annual temperature and conductivity of leachate from the two control sites, a polluted water stream in the vicinity of the waste tip and an unpolluted stream, differ significantly. However, only the temperature explained the differences in plant traits. In April, and in some cases in May, plants in the leachate were significantly higher than in those on the control sites in terms of biomass and plant height. Thermal pollution caused a phenological shift in all species and also caused *Scirpus* plants to die out more quickly. Temperature also affected the proportion flowering vs. vegetative individuals, e.g., none of *Scirpus* plants started to bloom.

## Introduction

In terms of worldwide mineral resources, the exploitation of coal is the most common (USGS 2005). The exploitation of this mineral leads to the production of large amounts of wastes. On average, there is 0.4 ton of wastes per 1 ton of coal mined. Due to their physicochemical properties, these kinds of wastes are not considered to be dangerous. However, they pose a threat to freshwater resources because of the many contaminations that occur in freshly produced wastes and those that are caused by the physical–chemical decline that is mobilized next and transported by a stream of infiltrated waters (Szczepańska and Twardowska 1999; Szczepańska and Twardowska 2004). Waste heaps that are still active (burning) which emit considerable amounts of gas pollutants into the air are especially dangerous to the natural environment. One characteristic trait of old post-colliery waste tips is their thermal activity. The capability of waste heaps to continue burning is a consequence of the high contribution of coal material which can be as high as 30 % of the total mass. The burning of a waste heap can result from both exogenic processes when it is initiated by external sources of heat, or endogenic ones, i.e., autonomous combustion as a result of the oxidation of substances, which is accompanied by emission of high amounts of heat (Falcon 1986; Chakravorty and Kolada 1988; Cebulak and Langier-Kuzniarowa 1997):

$$ {\mathrm{Carbon}} + {{\mathrm{O}}_2} \to {\mathrm{C}}{{\mathrm{O}}_2} + {\mathrm{heat}} $$

Endogenic burning is possible when the following factors are present: the presence of sufficient amounts of materials of appropriate activity relative to oxygen, easy access of air into the interior of the waste heap, and the possibility of heat accumulation in the heap, i.e., the rate of heap production exceeds the rate of heap emission (Szafer 1999).

In the present study, we introduce an example of a post-coal mine waste heap in which the processes of burning are still occurring. As a result of the burning of the waste heap, the temperature of the leachate, which originates from precipitation, is quite high, especially at the outflow. Modifications of the water temperature by humans are called “thermal pollution.” Most frequently, this phenomenon concerns the use of water by power plants and other industrial manufacturers for cooling (Prats et al. 2010) as well as urban runoff discharged into the surface waters from roads and parking lots (Anderson et al. 2010). While the physical and chemical properties of leachates and water quality in the vicinity of many landfills have been examined and reviewed in an extensive body of literature (Tałałaj and Dzienis 2007), there are no data that focus on its temperature and the thermal effect of the leachate on the water environment. There are a number of studies demonstrating the toxic effect of chemical substances in leachate on living organisms such as fish, algae, and invertebrates (Alkassasbeh et al. 2009). In order to fill this gap, we show the results of the impact of a warm leachate on chosen plant species. In the vicinity of the waste heap studied, the waters of the leachate flow into a small “river valley” that is surrounded by rush vegetation. Our main goal was to study the influence of thermally polluted water on the phenology and conditions of selected rush plant species.

The following research hypotheses were tested:

1. The growth of plants from the leachate is faster than plants from the control, i.e., they develop into the flowering and fruiting phases more quickly.
2. The percentage of flowering ramets in the leachate is lower than in the control.
3. Ramets decline faster in the leachate as a result of a phenological shift and/or an ecotoxicological effect of water temperature.
4. The selected species differ in their responses to thermal pollution.

## Material and Methods

### Study Area

**Fig. 1:**
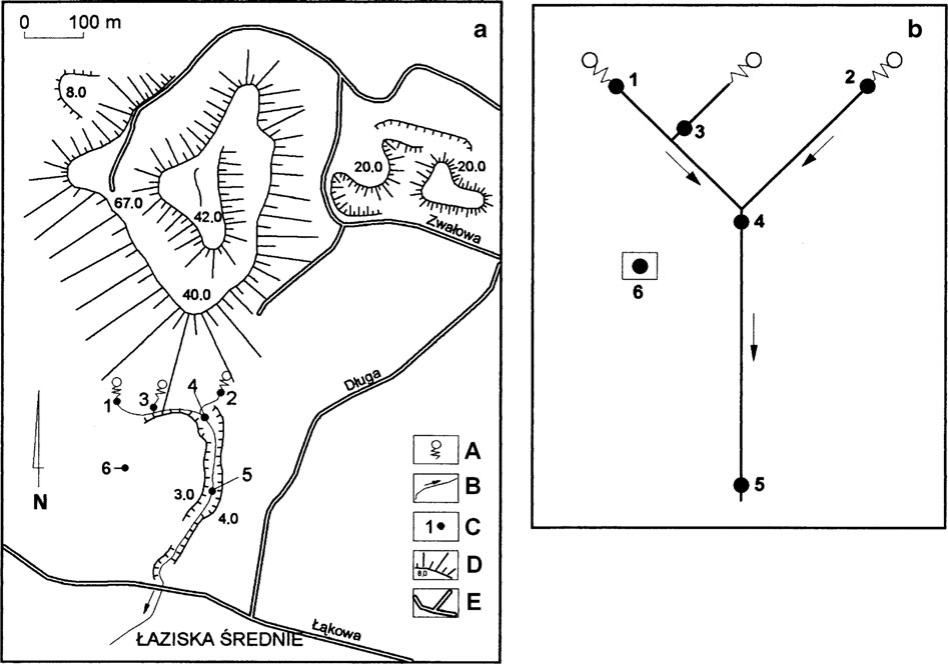
The study area (**a**) of the colliery waste tip “Skalny” and sampling design (**b**). *A* sewage water outflows, *B* sewage stream, *C* study plots (*1*–*6*), *D* slopes of waste tip, *E* roads

The colliery waste heap (waste heap “Skalny”) is located in southern Poland, in a mesoregion Katowice Upland, a part of Silesian Upland, on the territory of the town of Łaziska Średnie (50°8′27″ N, 18°51′19″ E, Fig. 1). This waste site is a hill with a relative height of 90 m. The area occupied by this waste heap is ca. 30 ha and the amount of wastes is estimated to be about 17 million Mg. The wastes were deposited in the years 1912–1998 (Woźniak 2010). Beginning in the 1960s of the twentieth century, the intense development of thermal processes, including burning, was observed. Land reclamation practices aimed at the liquidation of burning sites, which included the formation of slopes and biological building (sowing of grasses), were started in 1999. Due to the large mass of deposited wastes and former burnings, these thermal processes cannot be expected to be reduced quickly. There are sewage water outflows close to southern edge of the waste heap. The origin of sewage waters is caused by small river valleys being filled with post-coal mine wastes. Nowadays, infiltration waters are captured by a “drainage system” of buried river valleys and then they are transferred through the main valley outside the waste heap (Fig. 1).

The outflows of sewage waters are permanent; they function throughout the whole year. A summary yield of all outflows ranges from 0.5 to 1.5 dm^3^/s.

### Sampling Design

In total, seven study plots were chosen in 2011. They varied in shape and size from 2.25 m^2^ (1.5 × 1.5 m) to 25 m^2^ (5 × 5 m) depending on the presence of vegetation. The study plots encompassed the stream zone and the banks of a particular watercourse. Five study plots were established within the anthropogenic river valley of sewage water (leachate). The first two plots were on the sites of sewage outflows; the remaining ones were laid out within a longer, more than 100 m, section of this anthropogenic water stream (Fig. 1). The sixth plot was set in a water stream flowing through meadows in the vicinity of the waste tip. This plot served as a first control site and is referred to as a polluted water stream. In order to eliminate the effects of the neighborhood of the waste tip and possible chemical pollution, a seventh plot was also established in the vicinity of an undisturbed water stream, a tributary of Mleczna River, which is considered as a second reference sample (control). It is situated in the same geographical region (Katowice Upland)—in a southern, suburban part of the city of Katowice (50°12′15″ N, 18°57′38″ E). Mleczna is small river (length is ca 22 km and the area of catchment amounts to 142 km^2^). The temperature and conductivity of waters were measured twice a month from plots 1–7. In addition, from plots 3–5 and 6 and 7 data on the morphometric traits of three species, common bulrush *Typha latifolia*, common reed *Phragmittes australis*, and wood club-rush *Scirpus sylvaticus* were collected from water stream and reference samples, respectively. The measurements were carried out in April, May, June, August, and November. Next, the number and percentage of various stages of these plants were counted. The shoot height, number of leaves, and width of leaves were measured in all present individuals. From five to seven plants of each species were removed and dried for 48 h at 60 °C. The total biomass of the dried individuals was weighed.

### Statistical Analyses

To compare the mean annual temperature and conductivity of waters between the study plots over time, the Friedman test followed by Siegel–Castellan procedure were applied (Siegel and Castellan 1988). The difference in the frequency of flowering plants vs. vegetative plants was analyzed using the *G*-test both for the comparison of species and for the comparison of leachate and polluted water streams. The flowering and barren plants were counted separately for each species during the most favorable period for blooming. The differences in plant traits (biomass, shoot height, number of leaves, and width of leaves) for each species between the types of water (leachate, water stream, and control) were examined separately using the Kruskal–Wallis test, and in cases where this test indicated significant differences, their medians were compared using the Steel–Dwass test in particular months. In the case of an absence of plants in a specific period, usually at the control site, the Mann–Whitney *U* test was used. All statistics were calculated using R software (R Development Core Team, 2009).

## Results

### Characteristics of the Waters of the Leachate and Wetland in the Vicinity of the Waste Tip

The mean annual temperature on the first study plot (outflow at the slope of the waste tip) was the highest and amounted to ca. 50 °C during the course of the year, whereas on the second study plot, it was around 30 °C (Table 1). The outflow temperature of the water decreased significantly with the distance from the source down to the level of ca. 18 °C. On next three study plots, the decrease in the temperature was statistically significant in comparison with the two reference study plots (Table 1). The mean annual conductivity of the leachate was similar in all of the study plots amounting to around 13–14 mS/cm with the exception of the sixth and seventh study plots where on average 9 and 0.6 mS/cm, respectively, was recorded (Table 1).

**Table 1:** Average annual temperature (means ± SD) and average annual conductivity of the leachate (nos. 1–5), water stream (6), and control (7) in the study plots

Number of study plot	Temperature (°C)	Conductivity (mS/cm)
1	49.75 ± 0.26 a	13.36 ± 0.64 a
2	30.92 ± 0.76 b	13.30 ± 1.00 a
3	16.58 ± 1.49 c	13.05 ± 0.27 a
4	14.25 ± 6.55 c	13.89 ± 0.85 a
5	13.5 ± 6.94 c	13.8 ± 0.9 a
6	9.25 ± 8.31 d	8.82 ± 0.52 b
7	10.25 ± 7.61 d	0.61 ± 0.09 c

The values with different letters in a column differ significantly at the *p* level <0.05 (Friedman test followed by Siegel–Castellan test)

### Comparison of the Percentage of Generative Ramets Between the Species and Water Source

**Fig. 2:**
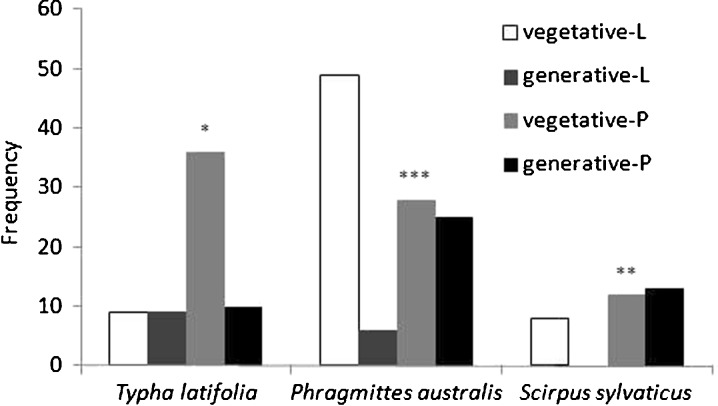
Differences in the proportion of generative and vegetative ramets between the leachate (*L*) and polluted water stream (*P*) in the vicinity of the waste tip for particular species (*G*-test *p < 0.05, **p < 0.01, ***p < 0.001)

A comparison of the proportions of generative and vegetative ramets in focal species revealed significant differences between the leachate and the water stream sites (Fig. 2). The percentage of flowering ramets (50 %) in *T. latifolia* growing in the leachate was higher than in the vicinity of the polluted water stream (21.7 %), whereas the percentage of flowering ramets (10.9 %) in *Phragmites australis* in leachate was much lower when compared to the polluted water stream (47.1 %). As was mentioned earlier, no flowering plants in *S. sylvaticus* were observed in the leachate, whereas in the polluted water stream 52 % of ramets were in the bloom phase (Fig. 2).

### Comparison of the Traits of the Helophytes Due to Water Source

**Fig. 3:**
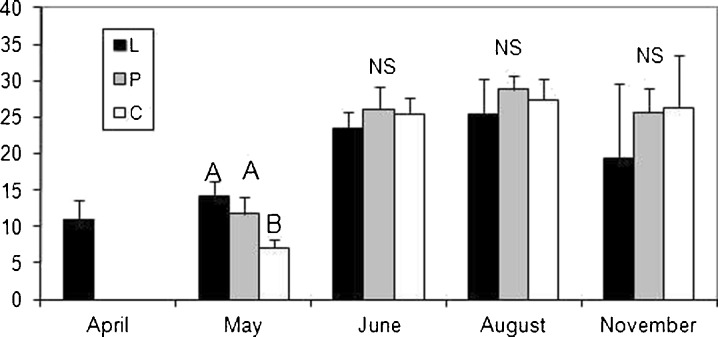
Comparison of the biomass (in grams) in *T. latifolia* between the sites studied (means ± SD). Abbreviations: *L* leachate, *P* polluted water stream, *C* control. *Bars with different letters* in a given month differ significantly at the *p* level <0.05, *NS* nonsignificant (Kruskal–Wallis test followed by Steel–Dwass test)

**Fig. 4:**
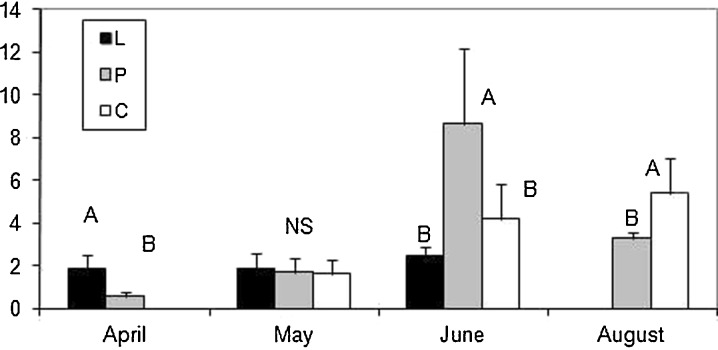
Comparison of the biomass (in grams) in *P. australis* between the sites studied (means ± SD). Abbreviations: *L* leachate, *P* polluted water stream, *C* control. *Bars with different letters* in a given month differ significantly at the *p* level <0.05, *NS* nonsignificant (Kruskal–Wallis test followed by Steel–Dwass test)

**Fig. 5:**
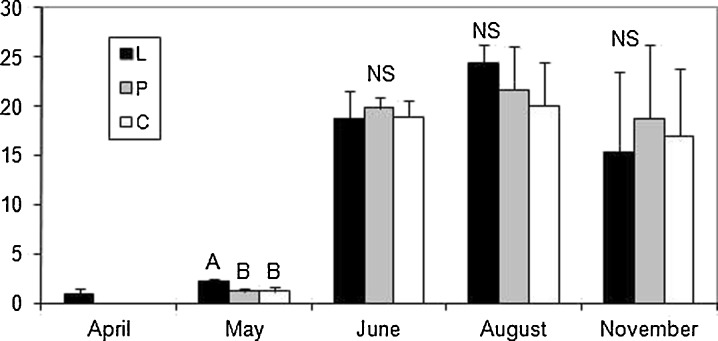
Comparison of the biomass (in grams) in *S. sylvaticus* between the sites studied (means ± SD). Abbreviations: *L* leachate, *P* polluted water stream, *C* control. *Bars with different letters* in a given month differ significantly at the *p* level <0.05, *NS* non-significant (Kruskal–Wallis test followed by Steel–Dwass test or Mann–Whitney *U* test)

In April, plants of *T. latifolia* and *P. australis* were present only in the leachate (Figs. 3 and 4); however, individuals of *S. sylvaticus* grew in both the leachate and in the polluted water stream, although they were not observed in the water of the control (Fig. 5). The majority of the significant differences in the selected morphometric traits were observed in May. There were only significant differences between the leachate and the polluted water stream in the biomass of *T. latifolia* (Fig. 3); however, all three groups of plants differed significantly in relation to the mean height of plants (Table 2). As to the mean number of leaves, individuals from the leachate had a significantly higher number of leaves than the remaining two groups (Table 2). There were only statistically nonsignificant differences in the width of leaves and in the above-mentioned parameters in later months. Significant differences were observed in *P. australis* only in April. The plants that grew in the leachate were taller (Table 2) and had a higher biomass in comparison to those that grew in the polluted water stream and control (Fig. 4). All three groups differed in the number of leaves, i.e., plants from the leachate had a higher number of leaves but plants from the control had the lowest mean number of leaves per plant.

**Table 2:** Comparison of the remaining plant traits of macrophytes between the three types of water

Month	Plant characteristics	Leachate	Polluted water stream	Control
April	Differences in plant height in *Typha latifolia* (cm)	89.18 ± 20.64	–	–
May		**122.57 ± 8.79 a**	**97.00 ± 20.03 b**	**63.63 ± 9.35 c**
June		191.43 ± 4.28	193.57 ± 3.60	192.00 ± 3.83
August		192.00 ± 16.36	208.57 ± 15.60	201.71 ± 20.52
November		140.75 ± 76.53	182.71 ± 18.46	184.63 ± 52.91
April	Differences in number of leaves in *Typha latifolia*	6.45 ± 1.81	–	–
May		**8.71 ± 1.11 a**	**6.71 ± 0.95 b**	**5.73 ± 1.01 b**
June		10.57 ± 1.27	10.71 ± 1.11	11.29 ± 1.25
August		10.00 ± 1.29	11.71 ± 1.70	12.14 ± 1.77
November		9.13 ± 2.70	9.86 ± 1.57	12.13 ± 3.60
April	Differences in width of leaves in *Typha latifolia* (cm)	17.60 ± 3.15	–	–
May		18.11 ± 1.76	19.31 ± 1.74	18.19 ± 2.03
June		20.66 ± 1.24	20.87 ± 2.05	20.56 ± 1.60
August		19.36 ± 2.19	21.36 ± 0.99	21.64 ± 1.95
November		17.31 ± 4.21	19.50 ± 2.08	20.50 ± 3.74
April	Differences in plant height in *Phragmites australis* (cm)	42.93 ± 11.75	–	–
May		**76.63 ± 11.12 a**	**62.50 ± 7.69 b**	**58.71 ± 10.32 b**
June		181.79 ± 27.16	192.43 ± 9.98	183.14 ± 16.19
August		237.00 ± 17.19	226.71 ± 17.77	219.00 ± 16.05
November		205.57 ± 32.74	204.00 ± 39.97	203.86 ± 29.20
April	Differences in number of leaves in *Phragmites australis*	2.73 ± 0.80	–	–
May		**9.13 ± 3.27 a**	**3.63 ± 0.92 b**	**2.44 ± 0.51 c**
June		12.93 ± 1.07	12.86 ± 0.90	13.14 ± 1.21
August		15.00 ± 0.58	14.14 ± 2.04	13.57 ± 1.90
November		14.00 ± 1.15	14.14 ± 1.21	14.29 ± 1.11
April	Differences in width of leaves in *Phragmites australis* (cm)	13.89 ± 3.37	–	–
May		16.20 ± 2.35	15.15 ± 2.36	15.84 ± 3.14
June		38.48 ± 5.73	40.73 ± 2.11	38.76 ± 3.44
August		41.86 ± 2.63	43.87 ± 0.91	42.43 ± 1.80
November		40.37 ± 3.10	39.70 ± 6.67	40.67 ± 3.42
April	Differences in plant height in *Scirpus sylvaticus* (cm)	**44.14 ± 14.00 a**	**17.78 ± 3.90 b**	–
May		44.25 ± 5.09	43.25 ± 8.75	41.75 ± 6.86
June		**65.00 ± 7.28 a**	**98.25 ± 12.77 b**	**64.91 ± 10.91 a**
August		–	92.00 ± 7.16	93.86 ± 2.41
April	Differences in number of leaves in *Scirpus sylvaticus*	8.00 ± 1.83	7.85 ± 1.86	–
May		8.88 ± 0.83	8.00 ± 1.07	8.30 ± 1.77
June		10.00 ± 1.41	11.38 ± 0.92	10.00 ± 2.14
August		–	**11.50 ± 0.84**	**12.43 ± 0.53**
April	Differences in width of leaves in *Scirpus sylvaticus* (cm)	**13.14 ± 1.20 a**	**5.91 ± 1.97 b**	–
May		14.45 ± 1.75	13.95 ± 2.68	14.16 ± 2.17
June		16.36 ± 3.78	17.26 ± 2.26	15.18 ± 2.30
August		–	16.15 ± 1.27	16.53 ± 0.42

Means ± SD are shown. Values in bold with different letters in a given row differ significantly at the *p* level <0.05 (Kruskal–Wallis test followed by Steel–Dwass test or Mann–Whitney *U* test)

Individuals of *S. sylvaticus* differed in biomass (Fig. 5) and height (Table 2) between the leachate and the polluted water stream in April, but they were absent in the control. The plants from the polluted water stream were the tallest (Table 2) in June and were characterized by a higher biomass (Fig. 5). In August, the plants from the control site had a higher number of leaves compared to plants growing in the polluted water stream and plants in leachate declined at all. In April, the leaves of the plants from the leachate were wider.

## Discussion

The colonization and succession of vegetation in post-mining wastelands can resemble analogous processes in seminatural and natural biotopes (Woźniak et al. 2011). These habitats often function as sites where there is a presence of rare and protected plant species due to leachate inflows, which lead to the formation of wetlands at the bottom of colliery waste tips (Chmura et al. 2011). In the present study, the leachate differed from the polluted water stream flowing in the vicinity of colliery waste tip and the control both in terms of temperature and conductivity. However, the annual temperature of water between the disturbed water stream and the control did not differ significantly. However, the mean conductivity of the disturbed water stream was higher than the conductivity of the control. The latter is an indirect measure of dissolved organic matter including contaminants. As the comparison of the morphometric traits of plants showed, only temperature seemed to influence differences in plants between sites, especially at the beginning of the vegetation season.

Temperature is one of the most important environmental factors that has an influence on plant performance. Small thermal oscillations occur on a diurnal basis, while there is a more pronounced variation across the seasons, which is particularly dramatic across the latitudes between the extremes of Arctic and tropical climates (Berry and Bjorkman 1980; Berry and Raison 1981).

Plant species native to different climatic regions may be genetically adapted to the prevailing temperatures (Björkman et al. 1974, 1975) and, as a result, physiological functions may be limited in a way that restricts the species’ distribution (Woodward and Williams 1987; Woodward 1987). In addition, phenotypic modifications may allow individuals to adapt to local or temporal variations in temperature acclimation (Berry and Bjorkman 1980). Morphological adjustments or changes in the pattern of biomass allocation can also improve plant performance under contrasting thermal regimes, especially when they lead to increased growth or competitive ability. In *Dactylis glomerata* for example, the relative growth rate and the rate of root-cell division showed an increased tolerance to lower temperatures in high-latitude populations (Eagles 1967; Creber et al. 1993). In addition, the diversion of assimilates into the base and roots of individual plants provide storage reserves, which can be quickly mobilized to produce photosynthetic tissue in spring (Eagles 1967). However, populations may differ in the timing of growth and this, combined with preferences for particular microsites, may minimize differences in tissue temperature between environments and thus reduce the need for adaptation or acclimation (Berry and Raison 1981).

Our study showed that man-made changes in temperature affected the phenology of species that are macrophytes. We found that annual temperature was the highest at the outflow of the leachate. Water temperature does not change annually there. In the stream, the temperature decreases and undergoes greater fluctuations during the course of the year, but on average, it is higher than in the two control samples. No individuals of the focal species were found on the first two sampling sites. Perhaps the temperature there was the highest for the plants. Unusually, hot water enhanced faster development of the aboveground parts of plants and caused shifts in phenological stages in all three focal species. We studied emergent macrophytes that are rooted in the bottom and that stand erectly out of the water directly above their roots. Therefore, these plants are not fully under the influence of hot water, especially in the latter part of the vegetation season when the aboveground parts of plants are well developed and stand erect out of the water surface. As a study by Fletcher et al. (2000) showed, a high temperature over 50 °C due to thermal discharges from nuclear reactions can affect the canopy coverage of macrophytes even after 7–13 years. However, floating and emergent macrophytes did not vary greatly between thermally disturbed and undisturbed sites. It is worth mentioning that before their study, the area receiving thermal discharges over three decades caused almost all of the animal and plant communities to die out and seriously damaged the adjacent riparian zone. The influence of thermal water pollution over time has a negative effect. In the present study, only plants of *S. sylvaticus* seemed to be affected by hot water, which was reflected by a lower biomass in June and their decline in August. In the remaining two species, biomass and other morphometric traits revealed lower values but there were nonsignificant differences between the water stream and the control. In addition to high temperature, water contamination, i.e., a high content of phosphates, may influence the development of plants. An increased concentration of nutrients can also have an impact on the phenology and growth of the aboveground parts of plants. In our study, only in *S. sylvaticus* were individuals present in the water stream in August. The waters there are more contaminated as compared to the control. Moreover, there were no significant differences in biomass between the leachate and the water stream in *T. latifolia* in April.

## Conclusions

To sum up, we can conclude that thermally polluted water generally does not negatively influence the occurrence and condition of macrophyte vegetation. Species which build such communities like the common reed, common bulrush, and wood club-rush are regarded as widely tolerant and therefore the discharge of hot water only caused a shift in their phenology. Further research is required in order to examine the negative effects cause on the development of vegetation over a longer period of time. Therefore, our study can be regarded as preliminary.
